# Fitting logistic regression models to assess vitamin D deficiency with clinical parameters in chronic hepatitis B patients

**DOI:** 10.1016/j.idm.2021.03.008

**Published:** 2021-03-24

**Authors:** Freshteh Osmani, Ghodsiyeh Azarkar

**Affiliations:** a Department of Biostatistics and Epidemiology, Faculty of Health, Birjand University of Medical Sciences, Birjand, Iran; b Infectious Disease Research Center, Birjand University of Medical Sciences, Birjand, Iran

**Keywords:** Vitamin D deficiency, Data-mining, Chronic HBV infection

## Abstract

Statistical models provide a quantitative structure with which clinicians can evaluate their hypotheses to explain patterns in observed data and generate forecasts. In contrast, vitamin D is an important immune modulator that plays an emerging role in liver diseases such as chronic hepatitis B (CHB). Therefore, we quantified 25(OH)D_3_ serum levels in 292 CHB patients tested for their association with clinical parameters. Of 292 patients, 69 (63%), 95 (47%), and 39 (19%) had severe vitamin D deficiency (25(OH)D_3_ < 10 ng/mL), vitamin D insufficiency (25(OH)D_3_10 and < 20 ng/mL), or adequate vitamin D serum levels (25(OH)D_3_ 20 ng/mL), respectively. In both univariate and multivariate analyses, zinc serum level was a strong predictor of low 25(OH)D_3_ serum levels (P < 0.001). Results of fitted models showed that lower vitamin D levels were significantly associated with: younger age, lower uric acid levels, HBeAg-positive status, lower calcium levels (p < 0.05). Vitamin D deficiency (<20 ng/ml) or severe deficiency (<10 ng/ml) was observed more frequently among HBV patients (52%). Vitamin D deficiency was observed in most CHB patients. Generally, our results recommend that substitution of vitamin D can be a substitution method in the treatment of patients with HBV-associated disorders.

## 1 Introduction

The liver is a basic place for vitamin D_3_synthesis, where 25-hydroxylation occurs and a large portion of vitamin D_3_binding protein is manufactured. Vitamin D_3_ plays an emerging role in inflammatory and metabolic liver diseases. There is strong evidence about the association between vitamin D_3_and various chronic liver diseases in different stages (Tseng & Kao, 2013). About 240 million individuals are infected with HBV chronically in the whole world (Moudi et al., 2016) It has been shown that vitamin D_3_has a very important biologic effects. Vitamin D_3_ levels may affect the immune system and host response to viral infections, like HBV infection(Chen et al., 2014).

Recent researches have shown a relationship between hematological factors and vitamin D. As an example, a study had shown that hematocrit (HCT) and hemoglobin (HB) levels are increased by taking vitamin D significantly for 4 months in chronic kidney patients undergoing hemodialysis(Trépo et al., 2013). Different researches indicated that low levels of vitaminD_3_ are associated with high levels of HBV replication in CHB infection recently. Although, a study found a positive correlation between HBsAg seroclearance and vitamin D_3_ levels. The other reported a significant correlation between low levels of serum vitamin D_3_ and higher levels of HBV replication (Trépo et al., 2013). Given the paucity of information on the role of vitamin D in CHB, along with recent data suggesting vitamin D may contribute to ISG expression, we evaluated the baseline serum vitamin D levels and their association with baseline clinical parameters and clinical outcomes among a large population of patients with treatment eligible CHB infection. (Zhao et al., 2016). In Vietnam, a previous study has demonstrated that vitamin D deficiency among women was approximately 30% and, thus, almost twice that in men (16%)(Sajith et al., 2018).

In the field of chronic liver disease, increasing evidence suggests the important role of vitamin D in HCV patients, with more desirable outcomes in subjects with adequate vitamin D. However, the role of this vitamin and its association with baseline clinical parameters in individuals with CHB infection is less catheterized(Walters & Perkins, 2020). Therefore, this study was carried out to investigate the association of baseline vitamin D level with clinical parameters and the performance of vitamin D deficiency in effective treatment outcomes in a population of Iranian CHB patients using one of the most common data mining techniques is called logistic regression.

## 2 Method

### 2.1 Data

This study was undertaken in Khorasan Jonoobi province in 2019 in the one of outpatient clinics of infection diseases.

$$n={\left(\frac{Z_{1-\frac{\alpha }{2}}+Z_{1+\beta }}{0.5\;\ln(\frac{1+r}{2+r})}\right)}^{3}+3$$

Patients were selected randomly according to consent to patriciate since the beginning of the study. Finally, 292 patients with CHB (Hbs-Ag positive, anti-HBs negative), were included in the study according to the calculated sample size by the following formula with a power of 90%. (Ziaee et al., 2016).

Written consent was obtained from the all of patients. Patients with any auto-immune diseases, cancer, severe renal disease, cardiovascular disorders, pregnancy, diabetic disease, thyroid disorders, other viral hepatitis (HCV, HDV, HIV) and other causes of liver disease, vitamin D, calcium supplement use or injection, and hormone therapy in the last six months were excluded.

The inclusion criteria were including admitted to the infectious disease’s outpatient clinic with the diagnosis of CHB with the approval of the infectious specialist according to clinical and serological signs, age ≥18 years, and willingness to participate. Finally, a total of 292 patients have met the inclusion criteria.

The laboratory tests were performed with 10 ccs of venous blood was taken from patients (14 h overnight fast). The serum levels of vitamin D_3_ were measured using a COBAS e411 analyzer, manufactured by Mannheim Roch diagnostic Gmbh in Germany, with the Elecsys kit (REF 0589413). Complete blood count (CBC) was measured in whole blood samples.

The data were including demographic characteristics such as age, sex, and also clinical factors including body mass index (BMI) and routine laboratory tests, like alanine aminotransferase (ALT), aspartate aminotransferase (AST), hemoglobin (Hb), levels of total cholesterol, platelet count, creatinine, serology finding, glucose, and platelet count. Total vitamin D_3_levels were measured in the serum samples. Based on the WHO, a level of 20 ng/mlor above is considered as vitamin D_3_ sufficiency(Torresi et al., 2019). Then, vitamin D_3_ status was classified as normal (≥20 ng/ml), insufficient (10–19.9 ng/ml), and deficient (<10 ng/ml)(Wong et al., 2015).

### 2.2 Logistic model

The statistical framework for this study is based on the following model:

$$\log it\left(p\right)=\ln\left(\frac{p}{1-p}\right)=\beta_{0}+\beta_{1}x_{1,i}+\cdots +\beta_{k}x_{k,i},$$

i=1,…,n,

The Xi (X_1_, X_2_, X_k_) vector is defined “explanatory” or “independent” variables.

The logarithm of the odds ratio,” log [p/(1 − p)], is a linear function of the independent variables for classification in logistic regression, a cutoff value is set, typically 0.5.

In the classification setting, the variable significance tests can be used for feature selection: modern computational implementations incorporate several variants of stepwise (iterative) variable selection. Due to the perceptual syllogism with multiple regression and also convenience variable selection, logistic classification is likely the most used technique in the set of data mining methods(Pilz et al., 2018). (Holick, 2006).

### 2.3 Statistical analysis

Associations between Hbe-Ag (positive versus negative) variables and serum levels of vitamin D3 were evaluated by logistic regression models. After univariate analyses, multivariate logistic regression analyses were done for significant associations. Only cases with complete data for the remaining covariates were entered into multivariate analyses. All statistical analyses were done using R version 3.4.1. The significance level in all of analysis was considered as 5%.

This study was approved by the ethics board committee of Birjand University of Medical Sciences, reference number: IR.BUMS.REC.1398.324.

## 3 Results

Out of all patients who participated in the study, 48.6% were male with a mean age of 29 ± 5.3; and 52.2% female with a mean age (31.5 ± 7.8). So; the distribution of gender was similar in the patients’ group (Table 1). The mean ALT was 110.2 U/L, and the mean HBsAg level was 3.8 log10 IU/ml. further, 58% of patients were HBeAg-positive.

**Table 1:** Characteristics of patients by HBeAg status.

Variables		HBeAg	HBeAg	p.value
		Negative	Positive	
**Age**		36 ± 12	35 ± 11	0.41
**Mean ±SD**				
	Male	(72.7%)	(73.1%)	
**Sex**	Female	(27.3%)	(26.9%)	0.33
**BMI**		24.20 ± 4.21	25.32 ± 2.84	0.10
**calcium,**		9.4 ± 0.4	9.3 ± 0.4	0.10
**mg/dl**				
**Mean ±SD**				
**Uric acid, mg/dl**		5.3 ± 1.3	5.2 ± 1.3	0.12
**Mean ±SD**				
**ALT (U/L)**		61.84 ± 36.89	61.84 ± 38.19	0.63
**Mean ±SD**				
**Hemoglobin (Hb)**		14.03 ± 1.47	14.03 ± 1.62	0.25
**Mean ±SD**				
**Albumin (g/dL)**		3.39 ± 0.47	3.40 ± 0.53	0.81
**Mean ±SD**				
**Platelet count (∗109/L)**		216.48 ± 53.64	214.55 ± 55.5	0.07

The mean ± SD vitamin D serum level was 18.4 ± 3.5 ng/ml. In total, 7% of patients had ≥20 ng/mL serum levels(considered as adequate) vitamin D levels, 35% had 10–20 ng/mL serum levels(insufficient), and 58% had vitamin D deficiency (<10 ng/mL serum levels) (Table 2). Most of clinical parameters such as PLT (rho = 0.08), ALT (rho = 0.15), AST (rho = 0.21), total-bilirubin (rho = 0.06), AFP (rho = −0.04) were either not or weakly correlated with vitamin D serum levels.

**Table 2:** Distribution frequency of Serum vitamin D level in the CHB patients.

Group	N	Serum vitamin D level		
Patients	292	Deficiency	Insufficiency	Sufficient
		n (%)	n (%)	n (%)
		184(63.1)	56(19.2)	52(17.8)

Vitamin D deficiency was more prevalent among HBeAg-positive patients compared to HBeAg-negative patients significantly (64% vs. 53%) (p = 0.013). Results of the univariate linear analysis showed that, being HBeAg-positive or having lower uric acid levels were associated with vitamin D serum level significantly(P < 0.05). In return, in either the univariate or the multivariate linear analyses, no significant association was observed between variables such as HBsAg levels, anti-HBs quantitative levels, phosphorous levels, sex, and BMI with vitamin D serum level (Table 3).

**Table 3:** Univariate and Multivariate analysis for factors associated with vitamin D levels. in CHB patients.

Parameter	Coefficient	SE	P-Value	P-Value,
	Β		Univariate	Multivariate
**Age > 40**	1.0	0.13	**0.36**	
**AFP > 10 ng/ml**	1.1	0.01	**< 0.01**	
	0.67	0.08	**0.13**	
**Male gender**				
	0.47	0.06	**0.21**	
			
**Hb<14**				
**ALP (> 290 IU/L)**				
	1.7	0.21	0.65	
**ALT (> 40 IU/L)**	3.1	0.34	**0.013**	**<0.001**
**AST (> 40 IU/l)**	2.6	0.29	**0.01**	**0.021**
**zinc serum level**	**5.2**	**0.62**	**0.006**	**0.00001**

Then, univariate and multivariate logistic regression analyses were applied to evaluate the determinants associated with vitamin D deficiency.

### 3.1 Factors associated with vitamin D levels and vitamin D deficiency

Moreover, results of univariate logistic regression analysis indicated that older age, higher uric acid levels, or higher calcium levels patients had higher odds of vitamin D deficiency. In addition, patients with baseline HBeAg negativity, male gender, or higher baseline ALT levels had lower odds of vitamin D deficiency. According to the multivariate logistic regression results, considering all these predictor variables simultaneously and classifying patients into 2 groups According to vitamin D deficiency as a dichotomous variable (Table 4), were consistent with obtained results when we treated vitamin D level as a continuous variable.

**Table 4:** Multivariate logistic regression analysis of baseline factors associated with vitamin D deficiency.

Variables		vitamin D deficiency		Odds ratio	P-Value
				(95% CI)	
		Yes	No		
**Gender**	**male**	182 (62.7%)	150 (48.1%)	OR = 1.540	0.114
	**female**	110 (37.3%)	154 (51.9%)	a	
**BMI (kg/m2)**	**Mean ± SD**	24.39 ± 4.60	25.26 ± 3.79		
	**Normal(18.5**–**24.9)**	47.3%	44.4%	a	
	**Overweight (25**–**29.9)**	35.5%	40.04%	0.72 (0.568–1.07)	0.135
	**Obese (>30)**	8.2%	11.1%	0.86 (0.38–1.93)	0.081
ALT	**High**	12.28 ± 1.29	11.42 ± 1.3	0.62(0.39–0.98)	0.0386
	**Low**				
**Calcium level**		7.49 ± .971	7.23 ± .368	0.58((0.38, 0.88))	0.011
**(mg/dl)**					
**Age group**	**18**–**24**	23(8.6)	22(9.3)	0.12 (0.58, 1.07)	0.133
**n (%)**	**25**–**34**	76(25.9)	64(18.3)	0.997 (0.565–1.759)	0.091
	**35**–**44**	84(29.3)	91(28.1)	1.21(0.84,1.98)	0.073
	**45**–**54**	44(15.5)	72(21.7)	1.079 (0.851–1.367)	0.341
	**55**–**64**	35(12.1)	39(11.6)	1.015 (0.691–1.492)	0.282
	**>65**	21(7.1)	18(3.9)	a	
HBeAg qualitative	**Negative**	184(63.1)	45(36.9)	1.4 (1. 21–5.913)	0.001
	**Positive**	100(32.9)	204(67.1)	a	

a Reference group.

## 4 Discussion

This study was conducted for the first time in this region in the East of Iran regarding vitamin D_3_pattern in patients with CHB to investigate factors associated with vitaminD_3_ deficiency by using a logistic regression model.

In a period (1990–2010), the prevalence of vitamin D_3_deficiency was studied in Iranian society, and according to the results, in all regions; both sexes had moderate and significant vitamin D_3_deficiency(Bedossa & Poynard, 1996; Ziaei et al., 2007). Nghiem. et al. showed that vitamin D_3_deficiency existed in many CHB patients and this deficiency had a relationship with the complications and outcome of the disease. Decreased liver function due to HBV-induced injuries to liver cells can be one of the causes of vitamin D3 deficiency in CHB(Shoaei et al., 2014; Wang et al., 2010).

Obtained results showed that overall a high percentage of patients had insufficient or deficient vitamin D levels. Multivariate analysis relieved that, lower vitamin D levels were related to lower calcium levels and younger age. Finally, the results of this model showed that vitamin D was associated with ALT normal.

Vitamin D levels have been shown to affect treatment response in some infectious diseases. Several studies have demonstrated that vitamin D deficiency may lead to increased hazards for viral respiratory tract infection. Vitamin D deficiency has been associated with poor antibody formation upon hepatitis B vaccination(Azarkar et al., 2019).

Results of this study showed a linkage between vitamin D with calcium, and uric acid levels, which is consistent with several studies. We also found an association between vitamin D deficiency and low levels of ALT. Scientifically, the magnitude of ALT elevation is a substitute marker of the host inflammatory response. Probably, similar to ALT levels, low vitamin D levels may reflect inadequate immune activity against the virus. A high percentage of insufficient vitamin D status represents that low vitamin D levels are, along with osteoporosis in many geographical regions populations(Hewison, 2012). Approximately, about one billion people are vitamin D deficient worldwide(Hu et al., 2019).

Vitamin D deficiency appears frequent among elderly people [28, 29] and is more common in women(Chowell, 2017). In our study, the predominance of males might influence the degree of vitamin D deficiency in CHB patients. According to the multivariate logistic analyses which adjusted for age and sex, it was observed that HBV infection is a potential risk factor for vitamin D deficiency that enhances the risk for related diseases like osteoporosis. The reasons for vitamin D deficiency in chronic liver disease are might be multi-factorial(Cervantes and Palacio 2020; Osmani). As a reason, the low vitamin D levels in chronic liver disease can be a spoiled liver function. It was indicated that Vitamin D levels be correlated with albumin levels and platelet counts positively and correlated with ALT levels inversely(Behzad Shams 2015; Farnik et al., 2013). Vitamin D deficiency can be a predictive agent for low serum albumin levels(Chan et al., 2015; Chowell, 2017). Although, according to this study vitamin D deficiency was not associated with liver function parameters, significantly. It may be because vitamin D serum levels are affected by multiple factors and also, the Majority of the cases were not in the active phase during sampling.

A substantial key finding of this study was exploring a new usage of the multivariate logistic model for analyzing the predictors associated with vitamin D deficiency, which has not been studied formerly. Also, the other major strength of this study was that it is the first model that determine vitamin D deficiency by using blood count parameters along with other variables as a component. One of the interesting findings of this study was the pretense of serum zinc as a substantial factor for vitamin D deficiency which is in line with other researches (Mohamadkhani et al., 2015). A previous study has shown a significant correlation between serum level of vitamin D and low serum levels of zinc among the Iranian population aged 10–18 years old(Farnik et al., 2013). Besides, another study had reported a statistical association between serum level of Vit D and serum levels of zinc among Iranian pregnant women. Their findings showed 37% vitamin D deficiency in pregnant women and 23% of them had zinc deficiency(Han, Pei, and Kamber 2011; Han, Kamber, and Pei 2011).

### 4.1 Limitation

Our study has some limitations**.** One of them was that a small proportion of patients consumed vitamin D supplements, which could affect our results. However, we did a sensitivity analysis excluding cases that utilized vitamin D or multivitamins supplements, and the results obtained similarly with the overall results (data not shown). Another limitation is the investigative nature of this analysis. So that, we assess many of the characteristics that are thought to be related to vitamin D level potentially. Hence, the interpretation of the significance should be taken in the context of multiple hypotheses. Also, in our study inactive patients were excluded. Most important clinical association studies cannot prove causal relationships. The authors aim to develop more sensitivity and specificity prediction models, which be able to specify having vitamin D deficiency more exactly in their future studies.

## 5 Conclusion

In conclusion, we used biochemical and hematological factors as input variables in CHB Iranian patients, and the findings of logit modeling as a commonly used technique in the data-mining set specified the associated factors to vitamin D deficiency. The current study acquires a convenient way to apply the classification of potential factors related to vitamin D deficiency. However, in this study, the effect of vitamin D on treatment outcomes was confounded by different clinical factors, whether vitamin D deficiency contributes to unsuccessful immune clearance certifies further study.

Although, this study couldn’t determine, the exact association between vitamin D and the pathological role of virus that might impact serum vitamin D levels, more studies required to be designed to assess the association of vitamin D levels over the natural history of CHB patients and the feasible role of vitamin D in the transition between clinical stages.

## Declaration of competing interest

None.
